# Numerical analysis of an optical nanoscale particles trapping device based on a slotted nanobeam cavity

**DOI:** 10.1038/srep35977

**Published:** 2016-10-27

**Authors:** Senlin Zhang, Zhengdong Yong, Yaocheng Shi, Sailing He

**Affiliations:** 1 Centre for Optical and Electromagnetic Research, Zhejiang Provincial Key Laboratory for Sensing Technologies, JORCEP, East Building #5, Zijingang Campus, Zhejiang University, Hangzhou 310058, China; 2 Royal Institute of Technology (KTH), S-100 44 Stockholm, Sweden

## Abstract

A slotted nanobeam cavity (SNC) is utilized to trap a polystyrene (PS) particle with a radius of only 2 *nm*. The carefully designed SNC shows an ultrahigh Q factor of 4.5 × 10^7^ while maintaining a small mode volume of 0.067(*λ*/*n*_*water*_)^3^. Strongly enhanced optical trapping force is numerically demonstrated when the 2 *nm* PS particle is introduced into the central, slotted part of the SNC. In the vertical direction, the numerical calculation results show that a trapping stiffness of 0.4 *pN*/(*nm* · *mW*) around the equilibrium position and a trapping potential barrier of ~2000 *k*_*B*_*T*/*mW* can be reached. To our best knowledge, the trapping capability (trapping stiffness and trapping potential barrier) of the proposed structure significantly outperforms the theoretical results of those in previously reported work. In addition, the SNC system does not suffer from the metal induced heat issue that restricts the performance of state-of-the-art optical trapping systems involving plasmonic enhancement. Based on the proposed cavity, applications such as lab-on-a-chip platforms for nanoscale particle trapping and analysis can be expected in future.

Optical trapping has been investigated extensively ever since A. Ashkin’s pioneering work on the manipulation of dielectric microspheres and single cells using laser beams^1^,^2^,^3^. Relying on the gradient force near the focus of a laser beam, researchers were able to manipulate micron-sized dielectric particles and biological cells. The noninvasive nature and high precision of the technique have boosted the potential of optical tweezers in chemical^4^,^5^, physical^6^,^7^,^8^,^9^, and biological applications^10^,^11^,^12^. However, the inherent diffraction limit of these free-space systems is a serious obstacle to enhancing the trapping strength. According to Tlusty, T. *et al.*’s work^13^, the gradient force applied on a Rayleigh particle (diameter much smaller than the wavelength) can be approximately derived as: 
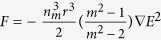
, where *n*_*m*_ is the refractive index of the surrounding medium, *r* is the radius of the particle, *m* is the ratio of the refractive indices of particle and surrounding medium and *E* is the electric field. Since the gradient force of small particles is proportional to the cubic of the radius of the particles, the diffraction limits the minimum possible volume of the matter to be manipulated (the radii of the particles are much smaller than the dimensions of the beam waist).

To deal with such an issue, research into near field optical manipulation techniques has been booming in recent years. Many proposed concepts such as driving particles in the evanescent field near a waveguide^14^, enhanced trapping force in slotted waveguides^15^,^16^, hybrid plasmonics waveguides^17^, optical microcavities^18^,^19^,^20^,^21^,^22^ and plasmonic bowtie systems^23^,^24^ have been demonstrated. These systems are able to further reduce the minimum size of the manipulated particles. For nanoscopic particles, these techniques fail to provide an adequate trapping potential barrier to localize the matter steadily unless very large optical power (up to several Watts) is injected according to the numerical analysis results of these references.

In this paper, we propose and numerically demonstrate a slotted nanobeam cavity to trap nanoscale particles firmly with quite low input optical power (as low as ~1 *mW*) injected into the waveguide. The ultra-strong optical confinement (deep subwavelength scale) in the slot region and ultrahigh-Q optical cavity contribute together to the state-of-the-art optical trapping capability for nanometric particles. The Q factor and mode volume of the proposed SNC are calculated to be 4.5 × 10^7^ and 0.067(*λ*/*n*_*water*_)^3^, respectively. Along the vertical direction, a trapping stiffness of 0.4*pN*/(*nm* · *mW*) around the equilibrium position and a trapping potential barrier of ~2000 *k*_*B*_*T*/*mW* (where *k*_*B*_ is the Boltzmann constant and *T* is the room temperature, 300 *K*) is achieved for a polystyrene particle (refractive index *n* = 1.59) with a radius of only 2 nm. The reason we choose a polystyrene particle is because the refractive index of this material is very similar to the refractive index of living cells or proteins. Thus, these particles can be used to simulate certain biological materials^16^. The calculation results show that the optical power as low as ~1 *mW* is enough to trap a 2 *nm* polystyrene particle. The theoretical trapping capability, which is characterized by the trapping stiffness and trapping potential barrier^15^, outperforms previous works^14^,^15^,^16^,^17^,^18^,^19^,^20^,^21^,^22^,^23^,^24^ remarkably. For example, in Allen H. J. Yang *et al.*’s typical work^15^, the theoretical trapping stiffness for a 50 nm particle is 0.2*pN*/(*nm* · *mW*). The exact trapping potential barrier value is not provided in the reference. However, given the relation between the trapping force and the position of the 50 nm particle^15^, the expected trapping potential barrier is also much smaller than that of the proposed structure if the input optical power is at the same level. In addition, the metal induced heat issue which occurs in the plasmonic optical trapping systems does not affect the proposed SNC system because it consists of only dielectric materials. Such a system may open a new realm of optical trapping of nanoscale particles for possible use in biological, chemical and physical fields.

## Results

**Figure 1:**
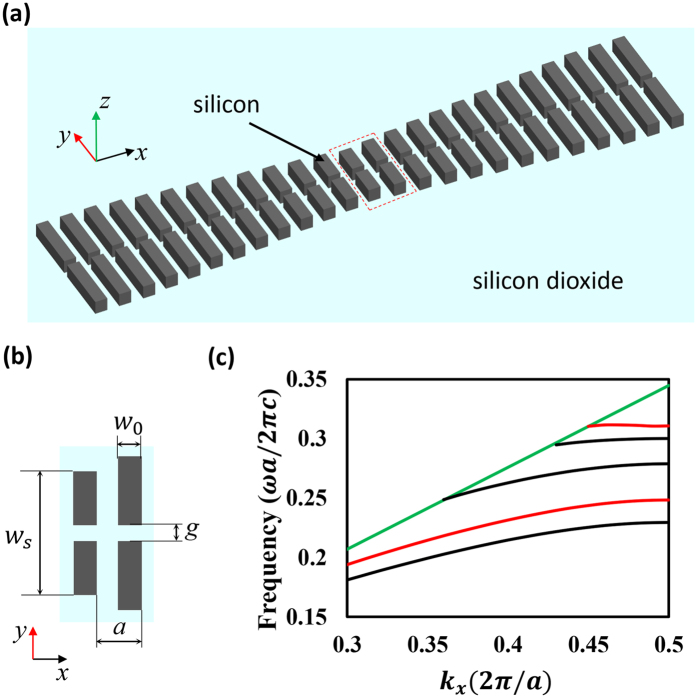
(**a**) The geometry of the slotted nanobeam cavity. (**b**) The top view of two cells enclosed in the red box in (**a**). The origin point of the Cartesian system is set as the center of the slot of the central stack. (**c**) The band structure of periodic stacks with *w*_*s*_ = 800 *nm* (red lines) and 1150 *nm* (black lines), respectively. The green line represents the light line.

The structure of the proposed SNC is shown in Fig. 1. Figure 1(a) shows the configuration of the system, in which the slotted nanobeam cavity is based on a standard silicon-on-insulator (SOI) chip. Quadratic width-modulated silicon stacks are designed according to the deterministic design process proposed by Quan Q. *et al.*^25^ to form a high-Q cavity. The quadratic modulated stacks are aimed at obtaining Gaussian attenuated mirrors from the center to the end. Such Gaussian shape mirrors are able to maximize the quality factor according to^25^. Figure 1(b) depicts the top view of two cells enclosed in the red box in Fig. 1(a). The geometrical parameters of the structure are as follows: the height of the silicon is *h* = 340 *nm*, the width and length of the silicon stacks are *w*_*0*_ = 160 *nm* and *w*_*s*_, which is quadratically increased from the center to the ends. The *i*^*th*^ stack’s length is defined as *w*_*s*_*(i)* = *w*_*s*_(*0*) + *i*^*2*^(*w*_*s*_(*N*) − *w*_*s*_(*0*))/*N*^*2*^, where *w*_*s*_(*0*) = 800 *nm* and *w*_*s*_(*N*) = 1150 *nm* are the lengths of the central and outermost stacks, respectively, and *N* = 15 is the number of modulated stacks in each side (including the central stack). Five stacks that are identical to the *N*^*th*^ stack are added at both sides to increase the Q factor of the SNC. The width of the slot is defined as *g*. The lattice constant of the cavity is *a* = 400 *nm*. The refractive index of silicon and silicon dioxide used here are 3.47 and 1.45, respectively. The system is immersed in water with a refractive index of 1.33. Figure 1(c) shows the band structure of periodic stacks with *w*_*s*_ = 800 *nm* (red lines) and 1150 *nm* (black lines), respectively. (The band structure is obtained through 3D Finite Difference Time Domain (FDTD)). The central point of the slot in the central stack is chosen to be the origin of the Cartesian coordinate system. The Cartesian coordinates system is depicted in Fig. 1(a).

**Figure 2:**
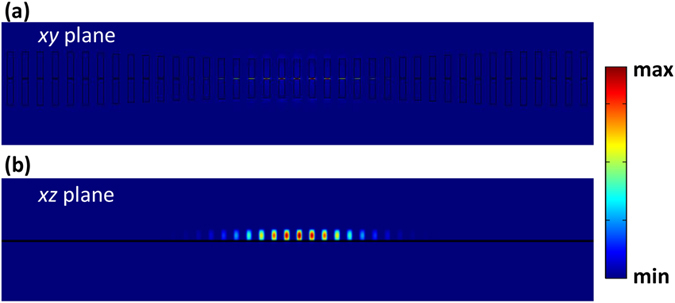
The electric intensity distribution (|*E*|^*2*^) of the SNC in the (*z* = 0 *nm*) plane (**a**) and (*y* = 0 *nm*) plane (**b**), respectively. The width of the slot is 20 *nm*. The resonant frequency is 184.33*THz*.

To investigate the resonance characteristics of the SNC, the Finite Element Method (FEM) based three-dimensional simulations are performed using commercially-available software (COMSOL Multiphysics). The calculated resonant profiles are shown in Fig. 2. Here, the width of the slot is 20 *nm*. Figure 2 (a,b) depict the |*E*|^*2*^distribution of the SNC in the *xy* plane (*z* = 0 *nm*) and *xz* plane (*y* = 0 *nm*), respectively. It is readily apparent that the optical electric field is well localized in the nanoscale slot. Such a deep-wavelength optical confinement will enhance the optical trapping capability dramatically, as discussed in the following sections^17^.

**Figure 3:**
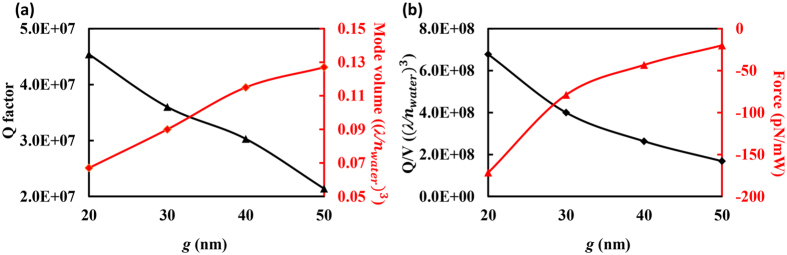
(**a**) The Q factor and mode volume of the cavities with different gaps. (**b**) The FOM (*Q*/*V*_*m*_) and trapping forces exerted on a dielectric particle with a radius of 2 *nm* when the gap of the cavity is changed. The particle is placed at (0, 0, 170) *nm*. The negative value of optical force corresponds to direction opposite the z-axis.

For such a slotted photonic crystal cavity, one important parameter that has a significant impact on the performance of the cavity is the width of the nanoscale slot (*g*). In order to study the dependence of Q factor and mode volume on the gap width, we performed a series of simulations in which *g* is varied. COMSOL Multiphysics is utilized here, and the calculated results are shown in Fig. 3(a).

The mode volume is evaluated as:


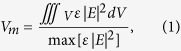


where *ε* is the relative permittivity, *E* is the electric field, and *V* is the calculation volume. According to Fig. 3(a), the Q factor decreases from 4.57 × 10^7^ to 2.14 × 10^7^ as *g* increases from 20 *nm* to 50 *nm* while the mode volume increases from 0.067(*λ*/*n*_*water*_)^3^ to 0.127(*λ*/*n*_*water*_)^3^. The Figure of Merit (FOM, i.e., *Q*/*V*_*m*_) decreases from 6.8 × 10^8^(*λ*/*n*_*water*_)^3^ to 1.7 × 10^8^(*λ*/*n*_*water*_)^3^ when *g* is varied from 20 *nm* to 50 *nm* as shown in Fig. 3(b).

Since the trapping capability of a cavity is partly determined by the FOM^26^, the relationship between the trapping force of a dielectric particle at a specified position and the FOM is also studied. To calculate the force exerted on a dielectric particle placed in the slot of the system, the Maxwell Stress Tensor (MST) *σ* is integrated around the surface of the particle: 
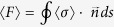
, where <*F*> is the time averaged optical force, *s* is the surface of the particle, and 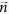 is the unit normal vector to the surface. The MST is expressed as:


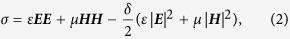


where ***E*** and ***H*** represent the electric field and magnetic field, *δ* denotes the unit dyad, and *ε*, *μ* are the permittivity and permeability of the surrounding medium, respectively^13^. To compare the forces that cavities with variable gaps exert on the target, a polystyrene particle (refractive index *n* = 1.59) with a radius of 2 *nm* is placed at (0, 0, 170) *nm*. The time averaged MST is evaluated using COMSOL Multiphysics, and the calculated optical forces are shown in Fig. 3(b). The dependence of the optical force on the FOM of the cavity can be clearly seen from Fig. 3(b), as the optical force shows a negative correlation with FOM (the force decreases quickly from −171.52 *pN*/*mW* to −19.96 *pN*/*mW*) when the gap is changed from 20 *nm* to 50 *nm*. (The negative value of optical force corresponds to the direction opposite the *z* axis.) Therefore, we focus on the cavity with a 20 *nm* slot and investigate its trapping performance.

**Figure 4:**
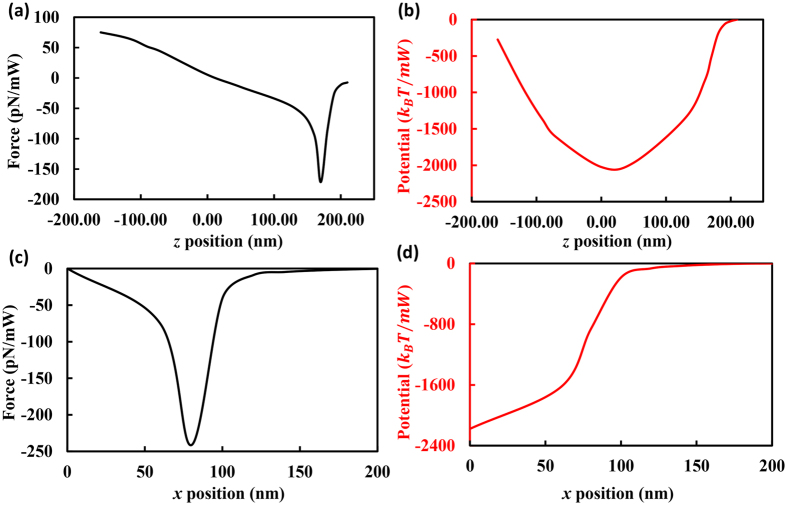
The variation of the trapping force and trapping potential when the dielectric particle with a radius of 2 *nm* moves along the *z* axis (**a,b**) and *x* axis (**c,d**).

The trapping capability of a system is characterized by the trapping stiffness and trapping potential barrier^15^. The trapping stiffness is defined as the variation of the optical force over unit distance away from the equilibrium position for the particle, and the trapping potential barrier is related to the kinetic energy required for the particle to escape from the trapping zone. For a SNC with a gap of 20 *nm*, the trapping capability is studied when the position of the aforementioned polystyrene particle is changed. Figure 4 shows the dependence of the trapping force and trapping potential on the position of the particle. Only the variations of optical force along the *x* and *z* directions are studied, as there is a physical barrier along the *y* direction for the slot which will prevent any movement of the particle^26^. Figure 4(a,b) show the trapping force and trapping potential of the particle when the position of the particle is changed from (0, 0, 0) *nm* to (0, 0, 210) *nm*. From Fig. 4(a), one can see that the particle experiences a trapping stiffness of 0.4*pN*/(*nm* · *mW*) around the equilibrium position. This value is significantly higher than previous systems that work for larger particles^15^,^16^,^17^,^18^,^19^,^20^,^21^,^22^,^23^,^24^. (See ref. 15 for an example, which reported a 0.2*pN*/(*nm* · *W*) trapping stiffness for a nanoparticle with a radius of 50 *nm*). To obtain the trapping potential, the trapping force is integrated along the *z* axis^27^, and the calculated results are shown in Fig. 4(b). If a power of 1 *mW* is injected into the system, a maximum trapping potential barrier of ~2000 *k*_*B*_*T* is achieved, which is much larger than the trapping potential depth threshold (10 *k*_*B*_*T*)^26^. This result means that the system can trap the 2 *nm* PS particle firmly along the vertical direction even with a much lower input optical power than 1 *mW*. When the particle is moving along the *x* direction (from (0, 0, 0) *nm* to (200, 0, 0) *nm*), the variations of the optical force and trapping potential are shown in Fig. 4(c,d). Along the *x* direction, the particle undergoes a trapping stiffness of 1.26*pN*/(*nm* · *mW*) around the equilibrium position, and a trapping potential barrier of ~2100 *k*_*B*_*T*/*mW* is achieved. Only the case where the PS particle has a positive *x* position is studied because the system is symmetric along the (*x* = 0 *nm*) plane (See Fig. 2), meaning that the shape of the optical force and potential in Fig. 4(c,d) are symmetric with respect to the origin point and the vertical axis (*x* = 0 *nm*), respectively. The trapping capability in the *x* direction is similar to that in the *z* direction, which means that the 2 *nm* PS particle can be trapped steadily by the proposed SNC system (In the *y* direction, the physical barriers will confine the particle in the slot region. In reality, the PS particle will be restricted in the central part of the slot in the *y* direction due to the symmetry of the electric intensity distribution about the (*y* = 0 *nm*) line^15^,^16^).

## Discussion

The proposed SNC is capable of trapping a nanoscale dielectric particle with a radius of 2 *nm*, which is not possible for conventional slot waveguides, hybrid plasmonic waveguides, and microcavities (such as photonic crystal cavities and microring resonators) when the optical power level is as low as that used in the proposed system. For structures exploiting plasmonic enhancement, trapping of particles with a radius of ~10 *nm* is possible^28^,^29^. However, these structures suffer from the following drawback: the optical power needed to trap such ~10 *nm* particles is about 10~20 *mW*, which may cause serious heat issues due to absorption of the metals in the structures that will break the trapping equilibrium^30^,^31^,^32^. Furthermore, to trap a sub-10 *nm* dielectric particle, the essential optical power will be scaled extensively according to the dependence of the trapping force on the volume of the target, meaning that the trapping balance will be broken as a result of the deteriorated heat issue. However, the proposed SNC is free from the metal-induced heat issue because the system consists only of dielectric materials (silicon and silicon dioxide). Additionally, the optical power needed to trap a 2 *nm* particle could be lower than 1 *mW*, which is much lower than required in the abovementioned structures to trap a much larger target. In summary, the proposed system outperforms the previous structures in that it can provide far superior optical trapping capabilities in the same optical input power level, and it does not suffer from the metallic structures induced heat problems.

What we should note here is that, although the calculation is performed for a dielectric particle, metallic particles can also be trapped steadily. This can be understood intuitively as follows: for small particles, the gradient force can be expressed as *F* ≅*V*_*p*_ *ζ* ∇|*E*|^2^, where *F* is the trapping force, *V*_*p*_ is the volume of the particle, and *ζ* is the relative difference of the permittivity of the particles and the surrounding medium^13^. Thus, given that *E* is affected negligibly by such a tiny particle, the trapping force of metallic particles shall be much bigger than that of dielectric particles due to the larger *ζ* for metallic particles than dielectric particles^16^.

## Conclusion

The trapping capability of a slotted nanobeam cavity is numerically investigated in this paper. For a polystyrene particle with a radius of 2 *nm*, the trapping stiffness around the equilibrium position is 0.4*pN*/(*nm* · *mW*), and the trapping potential barrier of the system is found to be ~2000 *k*_*B*_*T*/*mW*, which is sufficient to trap the target firmly. In order to evaluate the trapping performance of the proposed system, a detailed comparison of previous typical works and the proposed system is listed in Table 1. The results are all numerically evaluated numbers. Apparently, both the trapping stiffness and trapping potential barrier of the SNC is superior to the conventional, widely used structures. Besides, as we have discussed above, the SNC does not suffer from the metal-induced heat issue which impose restrictions on the applications of plasmonics enhancement involved systems. Moreover, the system is also capable of manipulating metallic particles. We believe the proposed SNC may be exploited for nanometric particle manipulation in biological, chemical and physical realms.

## Methods

The slotted nanobeam cavity is based on a standard commercial silicon-on-insulator chip. The thickness of the silicon is 340 *nm* and the quadratic width-modulated silicon stacks are designed referring to^25^. The refractive index of silicon and silicon dioxide are set as 3.47 and 1.45, respectively. The system is immersed in water with a refractive index of 1.33. The band structures are calculated using three-dimensional FDTD software (Lumerical FDTD solutions) in the design process of the slotted nanobeam cavity. Bloch boundary conditions are used in FDTD solutions to calculate the bandstructure. Specifically, the photonic bandstructure is calculated by determining the angular frequencies given the wave vector for all the Bloch modes in a given frequency range. The built-in bandstructure analysis groups of Lumerical FDTD solutions are used to find the accurate bandstructures. The optical performance of the cavity is obtained through the FEM-based eigenfrequency analysis method using COMSOL Multiphysics. To calculate the optical force, the time-averaged Maxwell Stress Tensor (MST), which is represented by the built-in Maxwell upward surface stress tensor in COMSOL Multiphysics, is integrated around the surface of the particle. The trapping stiffness of the system is derived by calculating the slope of the optical force-position curve (Fig. 4(a,c)) in the vicinity of the equilibrium position. The trapping potential is obtained through integrating the optical force along the *x* or *z* axis.

## Additional Information

**How to cite this article**: Zhang, S. *et al.* Numerical analysis of an optical nanoscale particles trapping device based on a slotted nanobeam cavity. *Sci. Rep.* **6**, 35977; doi: 10.1038/srep35977 (2016).

**Publisher's note**: Springer Nature remains neutral with regard to jurisdictional claims in published maps and institutional affiliations.

## Figures and Tables

**Table 1:** The trapping capability of typical optical trapping systems.

Structures	Trapping stiffness	Trapping potential barrier	Particle size (radius)
Slot waveguide^15^	0.2 pN/(nm*W)	/	50 nm
Hybrid plasmonic waveguide^17^	~30 fN/(nm*W)	6.2 k_B_T/W	2.5 nm
Nanoslotted photonic crystal cavities^18^	/	4 k_B_T/mW	10 nm
Silicon photonic crystal resonators^19^	8.5 pN/(nm*W)	/	50 nm
Tapered photonic crystal waveguide^21^	/	9.7 k_B_T/mW	50 nm
Gold bowtie plasmonic tweezers^24^	~60 pN/(nm*W)	9.79 k_B_T/mW	10 nm
Photonic/plasmonic cavity^26^	80 pN/(nm*W)	21 k_B_T/mW	20 nm
Coaxial Plasmonic Apertures^29^	/	0.6 k_B_T/mW	5 nm
Slotted nanobeam cavity	0.4 pN/(nm*mW)	2000 k_B_T/mW	2 nm

The numbers are all numerically evaluated.
